# Histological findings and NAFLD/NASH Status in liver biopsies of patients subjected to bariatric surgery

**DOI:** 10.20945/2359-4292-2022-0138

**Published:** 2023-11-10

**Authors:** Marielle Malucelli, Rodrigo Strobel, Claudia Ivantes, Danielle Sakamoto, Márcio Luís Duarte, Maria Lucia Alves Pedroso

**Affiliations:** 1 Universidade Federal do Paraná Departamento de Pós-graduação em Medicina Interna Curitiba PR Brasil Departamento de Pós-graduação em Medicina Interna, Universidade Federal do Paraná, Curitiba, PR, Brasil; 2 Pontifícia Universidade Católica do Paraná Curitiba PR Brasil Pontifícia Universidade Católica do Paraná, Curitiba, PR, Brasil; 3 Universidade Federal do Paraná Curitiba PR Brasil Universidade Federal do Paraná, Curitiba, PR, Brasil; 4 Universidade de Ribeirão Preto Campus Guarujá Departamento de Radiologia Guarujá SP Brasil Departamento de Radiologia, Universidade de Ribeirão Preto Campus Guarujá, Guarujá, SP, Brasil.

**Keywords:** Nonalcoholic fatty liver disease, nonalcoholic steatohepatitis, hepatic fibrosis, bariatric surgery

## Abstract

**Objective:**

To investigate nonalcoholic fatty liver disease (NAFLD), nonalcoholic steatohepatitis (NASH) and hepatic fibrosis in biopsies of people with obesity who underwent bariatric surgery and examine the possible association of different variables with a diagnosis of NAFLD and NASH.

**Materials and methods:**

Epidemiological, clinical and laboratory data from 574 individuals with obesity of both genders seen by the same physician between 2003 and 2009 who had a liver biopsy during bariatric surgery were examined.

**Results:**

Of the 437 patients included, 39.8% had some degree of liver fibrosis, 95% had a histologic diagnosis of NAFLD, and the risk factors were age ≥ 28 years and Homeostatic Model Assessment (HOMA) ≥ 2.5 (p = 0.001 and p = 0.016, respectively). In the NAFLD group, NASH was present in 26% of patients and the associated factors were aspartate aminotransferase and alanine aminotransferase index (AST/ALT) > 1, high-density lipoprotein cholesterol (HDL-c) < 40 mg/dL, total cholesterol (TC) ≥ 200 mg/dL, gamma-glutamyl transferase (GGT) > 38 U/L and triglycerides (TG) levels > 150 mg/dL. The independent risk factors were low HDL-c, elevated AST/ALT and high TG.

**Conclusion:**

The variables associated with a diagnosis of NAFLD were HOMA ≥ 2.5 and age ≥ 28 years. NASH was associated with low HDL-c, high TG and AST/ALT ≤ 1.

## INTRODUCTION

Nonalcoholic fatty liver disease (NAFLD) is defined as evidence of hepatic steatosis (HS), either by imaging or histology, and, absence of secondary etiologies of hepatic fat accumulation such as significant alcohol consumption, long‐term use of a steatogenic medications a fat concentration (1). It has a high global prevalence, estimated at 32% to 4%, and is currently recognized as one of the most common chronic diseases in developed countries (2–4). NAFLD is categorized by the spectrum of fatty liver disease which can be from simple hepatic steatosis or nonalcoholic fatty liver (NAFL) to nonalcoholic steatohepatitis (NASH), characterized by increasing hepatic fibrosis that eventually leads to cirrhosis, liver cancer, end-stage liver disease and death (1,2). Over time, the incidence and prevalence of NAFLD has dramatically increased, in parallel with the global epidemic of people with obesity, with histological changes similar to those caused by alcohol abuse, but as they do not involve alcohol, the term nonalcoholic is used (5–9). There are many risk factors for NAFLD, one of which is obesity, a pathological condition that is increasingly common in the general population (10). Indeed, approximately 40% of patients with NAFLD are overweight (11,12). Other risk factors for NAFLD are dyslipidemia (DSL), insulin resistance (IR) and type 2 diabetes mellitus (T2DM), indicating a link between NAFLD and metabolic syndrome (MS). Furthermore, patients with NAFLD have increasingly been found to present with IR (13,14).

The objective of this study was to evaluate NAFLD, NASH and hepatic fibrosis in biopsies of individuals with obesity who underwent bariatric surgery and examine the possible association of different variables with a diagnosis of NAFLD and NASH.

## MATERIALS AND METHODS

This study was conducted in accordance with the Declaration of Helsinki and was approved by the Scientific Committee and Research Ethical Commission of the Federal University of Paraná (Brazil). Informed consent was not needed, as the study was retrospective (Certificate of Presentation for Ethical Appreciation: 2191.085/2010-04).

### Patient characteristics

This retrospective cross-sectional study was carried out by reviewing the electronic medical records of 574 patients of both genres who underwent bariatric surgery performed by the same gastrointestinal surgeon at a private hospital in Brazil (located between 25° 25’ 17” south and 49° 17’ 26” west latitudes). The criteria for bariatric surgery, followed the criteria of the National Institutes of Health Consensus on Gastrointestinal Surgery for Severe Obesity (15). All patients had a body mass index (BMI) ≥ 40 kg/m^2^ or a BMI ≥ 35 kg/m^2^ with comorbidities, and all had been on an eating plan for at least 12 months.

All patients underwent a liver biopsy during the bariatric surgery, by the bariatric surgeon himself, with the purpose of verifying the existence or not of liver fat disease in the operated patients. All patients signed a consent form to perform this biopsy prior to the surgery and demographic, clinical, laboratory and histological data of the patients were collected.

### Inclusion and exclusion criteria

The inclusion criteria were as follows: (a) age over 15 years, (b) liver sample from perioperative biopsy available for review, and (c) specific clinical and laboratory data available for analysis. The exclusion criteria were (a) previous or current alcohol intake greater than 20 g of ethanol per day for women and 30 g per day for men, (b) presence of other liver disorders or viral, autoimmune, or hereditary liver diseases, (c) use of drugs that cause hepatic steatosis, or (d) specific clinical and laboratory data not available for analysis.

### Clinical, complementary exams and histological data

Patient anthropometric measurements (weight, height and BMI) were taken by the same dietitian. The following laboratory tests were performed preoperatively for all patients: gamma-glutamyl transpeptidase (GGT), aspartate aminotransferase (AST), alanine aminotransferase (ALT), glucose, insulin, total cholesterol, high-density lipoprotein cholesterol (HDL-c), serum triglycerides (TG) levels, and low-density lipoprotein cholesterol (LDL-c). In addition, 379 of the patients underwent ultrasound examination of the upper abdomen.

Patient data were analyzed for the presence of the following comorbidities: (a) DSL defined through the laboratorial classification of isolated increase in LDL-c (LDL-c ≥ 160 mg/dL); isolated increase in TG ≥ 150 mg/dL; increase in LDL-c (LDL-c ≥ 160 mg/dL) and TG (fasting TG ≥ 150 mg/dL or reduction in HDL-c (men < 40 mg/dL and women < 50 mg/dL) alone or in association with an increase in LDL-c or TG (16); (b) metabolic syndrome according to the International Diabetes Federation (IDF) definition (2006) (17); (c) a previous history of systemic arterial hypertension (SAH); (d) a history of T2DM and, for patients without diabetes, insulin resistance (IR) assessed by homeostatic model assessment-insulin resistance (HOMA-IR) using the formula: fasting insulin level (U/mL) x fasting glucose (nmol/L)/22.5 (18,19). The patient was considered to have IR whenever this index was greater than or equal to 2.5 (19).

All patients had an open liver biopsy performed at the end of their bariatric surgery to detect the presence or absence of chronic liver disease and allow for specialist treatment in the future. The liver biopsy samples were stained with hematoxylin-eosin, Masson's trichrome and Perl's Prussian blue and reviewed by the same pathologist. A simple steatosis diagnosis was made when there was steatosis infiltration > 5% hepatocytes with no or minimal inflammation, and NASH was diagnosed when steatosis, lobular inflammation and ballooned hepatocytes were observed, with or without fibrosis. The classification of NASH was carried out using the model proposed by Kleiner and cols. (20), and patients were assigned scores based on the presence of steatosis (0-3 points), lobular inflammation (0-3 points), ballooning (0-2 points) and fibrosis (0-4).

### Statistical analysis

Student's t-test was used to identify a possible association between the quantitative variables (age and BMI) and NAFLD or NASH. The chi-square test or Fisher's exact test was used to identify an association between NAFLD or NASH and the qualitative variables. To analyze the variables together, a logistic regression model was fitted, and the importance of each variable was assessed with the Wald test. ROC curve adjustment and the Youden criteria were used to determine the optimal cutoff value for age to discriminate NAFLD. P values < 0.05 were considered statistically significant. The data were analyzed with the Statistica v.8.0 program.

## RESULTS

### General characteristics of patients

**Figure 1:**
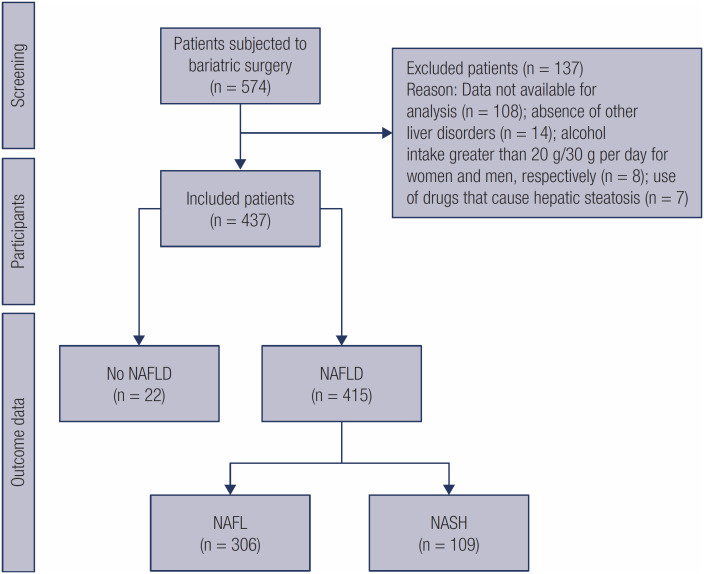
Description of patients in the study.

Of the 574 patients subjected to bariatric surgery, 437 were included, and of these, 415 (95%) had NAFLD and 109 (26.3%) also had NASH (Figure 1). Among the subjects included, the average age of patients with NAFLD was 36.5 years and the predominance of women was observed, with a greater number of cases with NAFLD being evident in both genders (Table 1). Among the evaluated comorbidities, there was a predominance of individuals with DSL, MS, SAH, T2DM, HOMA-IR ≥ 2.5.

**Table 1:** General characteristics of the patients studied

Variable	Classification	Results*
Age (years)	Mean	36,5 ± 10,6
BMI (kg/m^2^)	Mean	41,0 ± 4,8
Gender	Female	327 (74,8)
	Male	110 (25,2)
T2DM	No	344 (88,2)
	Yes	46 (11,8)
SAH	No	127 (32,6)
	Yes	263 (67,4)
MS	No	120 (40,1)
	Yes	179 (59,9)
Dyslipidemia	No	154 (39,2)
	Yes	239 (60,8)
HDL-c	<40 mg/dL	103 (28,5)
	≥40 mg/dL	210 (58)
	>60 mg/dL	49 (13,5)
LDL-c	<40 mg/dL	120 (40,7)
	>40 mg/dL	175 (59,3)
Total cholesterol (mg/dL)	<200 mg/dL	222 (59)
	≥200 mg/dL	154 (41)
GAMA-GT (U/L)	≤38 U/L	241 (72,2)
	>38 U/L	93 (27,8)
Glycemia	<100 mg/dL	262 (68,9)
	≥100 mg/dL	118 (31,1)
Insulin	<25 mU/L	231 (70,2)
	≥25 mU/L	98 (29,8)
HOMA-IR	<2.5	58 (18,1)
	≥2.5	263 (81,9)
AST/ALT	≤1	94 (25,7)
	>1	272 (74,3)
Triglycerides	≤150 mg/dL	211 (58)
	>150 mg/dL	153 (42)
Lobular inflammation	0	152 (45,2)
	1	95 (28,3)
	2	60 (17,9)
	3	29 (8,6)
Ballooning	0	180 (41,2)
	1	173 (39,6)
	2	84 (19,2)
Fibrosis	0	263 (60,2)
	1	88 (20,1)
	2	76 (17,4)
	3	8 (1,8)
	4	2 (0,5)
NAFLD ultrasound	No	136 (35,9)
	Yes	243 (64,1)
NAFLD biopsy	No	22 (5)
	Yes	415 (95)
NAFLD grade (biopsy)	0	22 (5)
	1	182 (41,6)
	2	139 (31,8)
	3	94 (21,5)
NASH	No	328 (75,1)
	Yes	109 (24,9)

* Data available for analysis

** Data were expressed as mean ± standard deviation or n(%).

BMI: body mass index;T2DM: type 2 diabetes mellitus; SAH: systemic arterial hypertension; MS: metabolic syndrome; HDL-c: high-density lipoprotein cholesterol; LDL-c: low-density lipoprotein cholesterol; GGT: gamma-glutamyl transferase; HOMA-IR: homeostasis model assessment of insulin resistance; AST/ALT: aspartate aminotransferase and alanine aminotransferase index; NAFLD: nonalcoholic fatty liver disease; NASH: nonalcoholic steatohepatitis.

Among patients with liver biopsy diagnosed NAFLD, 64.1% also received the same diagnosis through US, although 35.9% were not diagnosed as having NAFLD. Therefore, US sensitivity of 65.85% was observed for the diagnosis of NAFLD and specificity of 84.62% in this population studied. There was concordance between biopsy and ultrasound in the diagnosis of NAFLD in 66.5% of cases.

### Patients with NAFLD

Univariate analysis of all of the demographic, clinical and ultrasound data showed that there was a statistically significant association between the presence of NAFLD and age (mean 28.1 years for non-NAFLD and 36.9 years for NAFLD, p = 0.001). Gender (99.1% for males and 93.5% for females, p = 0.022) and the presence of dyslipidemia (94.2% for nondyslipidemia and 97.9% for dyslipidemia, p = 0.050) were associated with NAFLD (Table 2).

**Table 2:** Indicators for the presence of NAFLD

Variable	Classification	n	Patients with NAFLD	p value* (univariate analysis)	p value** (multivariate analysis)	OR (IC95%)
Age (y)	<28	111	98 (88.3)			
	≥28	319	311 (97.5)	<0.001	0.001	8.4 (2.4-30.0)
Gender	Female	327	306 (93.6)			
	Male	110	109 (99.1)	0.051	0.226	3.7 (0.4-31.9)
Dyslipidemia	No	154	145 (94.2)			
	Yes	239	234 (97.9)	0.050	0.852	1.1 (0.3-4.0)
Triglycerides (mg/dL)	≤150	211	197 (93.4)			
	>150	153	152 (99.3)	0.006	0.101	6.1 (0.7-53.3)
HOMA-IR	<2.5	58	51 (87.9)			
	≥2.5	263	256 (97.3)	0.005	0.016	4.4 (1.3-15.0)

* Fisher's exact test, p < 0.05.

** Logistic Regression Model and Wald test, p < 0.05.

HOMA-IR: homeostasis model assessment of insulin resistance.

**Figure 2:**
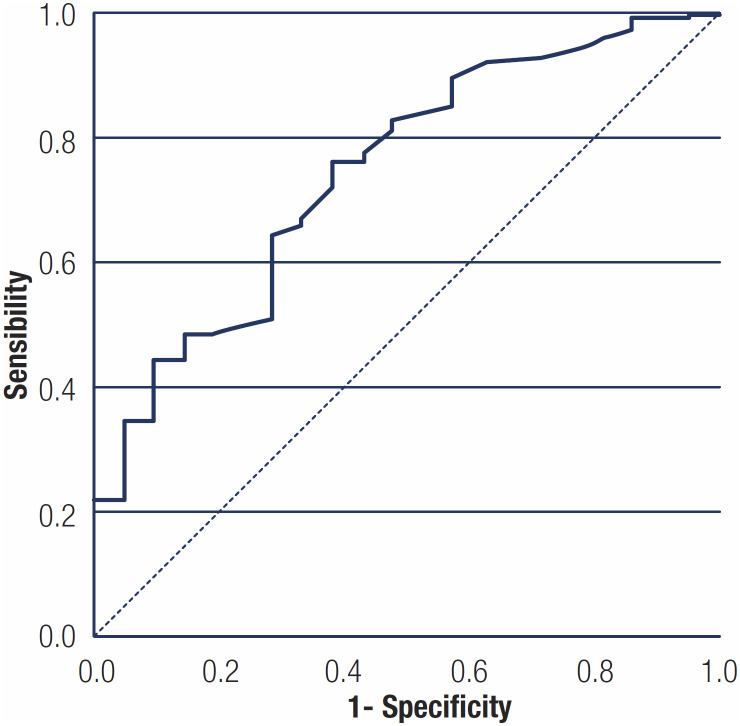
ROC curve of age predicting the presence of NAFLD.

The cutoff point for age determined by the ROC adjustment was 28 years (area under the curve 0.75, p < 0.001) (Figure 2), significantly associated with NAFLD regardless of the other variables included in the multivariate regression model (p = 0.001 and p = 0.016, respectively). The mean BMI of patients with NAFLD was 41 kg/m^2^, with no significant difference in relation to non-NAFLD (mean of 40.4 kg/m^2^, p = 0.599).

Among patients whose liver biopsy diagnosed NAFLD, 63.6% received the same diagnosis by US. However, 33% were not diagnosed by ultrasound. The results of the laboratory tests showed that there was a significant association between elevated triglycerides and HOMA ≥ 2.5 and NAFLD.

### Patients with NASH

There was a predominance of women (68%) among the patients with NASH, and the mean patient age and BMI were 37 years and 41 kg/m^2^, respectively. There was a statistically significant association between failure of NAFLD to NASH and ALT (77.2% for normal ALT and 58.6% for high ALT, p = 0.002). The factors associated with NASH in patients with NAFLD are shown in Table 3 (AST/ALT > 1; HDL-c < 40 mg/dL; TC ≥ 200 mg/dL, GGT > 38 U/L and TG > 150 mg/dL). Low HDL-c, elevated AST/ALT and elevated triglycerides were risk factors for NASH independent of the other variables.

**Table 3:** Characteristics of patients with NAFLD and NASH

Variable	Classification	N	Patients with NASH	p value* (univariate analysis)	p value** (multivariate analysis)	OR (IC95%)
AST/ALT	≤1	91	16 (17.6)			
	>1	262	76 (29.0)	0.037	0.032	2.3 (1.1-5.1)
HDL (mg/dL)	≥40	246	55 (22.4)			
	<40	102	36 (35.3)	0.016	0.050	1.9 (1-3.4)
Total cholesterol (mg/dL)	<200	210	44 (20.9)			
	≥200	151	54 (35.8)	0.003	0.066	1.8 (0.9-3.4)
GAMA-GT (U/L)	≤38	233	55 (23.6)			
	>38	90	33 (36.7)	0.025	0.290	1.4 (0.7-2.7)
Triglycerides (mg/dL)	≤150	197	35 (17.8)			
	>150	152	58 (38.2)	<0.001	0.002	2.7 (1.4-5.1)

* Fisher's exact test, p < 0.05.

** Logistic Regression Model and Wald test, p < 0.05.

AST/ALT: aspartate aminotransferase and alanine aminotransferase index; HDL-c: high-density lipoprotein cholesterol; GAMA-GT: gamma-glutamyl transferase.

### Histological evaluation

Among patients with NAFLD, 241 had no degree of fibrosis (F0), 88 (36.5%) had a level of fibrosis (F1), 76 (31.5%) had moderate fibrosis (F2), 8 (3.3%) had advanced fibrosis (F3) and 2 (0.8%) patients had cirrhosis (F4) (Table 4). Among the individuals with NASH, 51 (46.8%) had no degree of fibrosis (F0), 25 (23%) had a degree of fibrosis (F1), 29 (26.6%) had moderate fibrosis (F2), 4 (3.6%) had advanced fibrosis (F3) and none patient had cirrhosis (F4).

**Table 4:** Results of liver biopsies of patients with and without NAFLD and the presence or absence of NASH

Histological Findings		NAFLD Patients (n = 415)*		No NAFLD Patients (n = 22)*
		No NASH (n = 306)	With NASH (n = 109)	
Fibrosis				
	0	190	51	22
	1	63	25	0
	2	47	29	0
	3	4	4	0
	4	2	0	0
Ballooning				
	0	166	0	12
	1	124	41	10
	2	16	68	0
Lobular inflammation				
	0	130	0	20
	1	76	20	1
	2	11	48	1
	3	0	29	0

* Results expressed in numerical values. NAFLD: nonalcoholic fatty liver disease; NASH: nonalcoholic steatohepatitis.

An analysis of the correlation between the degree of fibrosis and the results of laboratory tests in patients with NASH could not be performed because there was a predominance of cases without fibrosis or with only mild fibrosis.

## DISCUSSION

The prevalence of NAFLD and NASH requires that different factors be taken into account, including (a) the geographic region where the study was conducted; (b) the criteria used to diagnose the condition, i.e., the serum aminotransferase levels, imaging results or histopathology; and (c) the characteristics of the study population, such as the general population or a population with risk factors (21,22). Estimates based on imaging and autopsies suggest that 20% to 30% of adults in the United States and Western countries have NAFLD (23). The prevalence is believed to be increasing and is even higher in at-risk populations (23). Studies on the prevalence of NAFLD and NASH using histological diagnosis are therefore needed.

Individuals with NAFLD often present with a higher BMI. A previous study showed NAFLD in 91% of people with obesity, 67% of overweight individuals and 24.5% of normal weight individuals (24). The prevalence of NAFLD in this population is particularly high (84% to 93%) (23), as in the present study (95%). However, the number of cases that evolve to NASH is not as high (23,25,26), which agrees with the findings of this study (26.3%).

Although initial observations suggested that NAFLD was more common in women, recent studies have shown slightly higher prevalences in men. The prevalence of NAFLD in women has been found to increase mainly during the postmenopausal period. It is believed that estrogen plays a role in the accumulation of fat in the gluteal-femoral region and that a reduction in these hormone levels during menopause contributes to the accumulation of abdominal fat. In men, the tendency to accumulate fat in the central region of the body is present in all age groups (1,27,28). In the present study, there was also a significant predominance of men with NAFLD, and the majority of women with NAFLD had not reached menopause.

NAFLD prevalence has been increasing in all age groups. However, it is most common in adults and tends to increase with age even in the absence of known risk factors for steatosis (29). A previous study analyzing middle-aged and elderly patients found a high prevalence of NAFLD (30). In this study, the mean age of patients with NAFLD and NASH was 37 years, which can be explained by the fact that this population consisted of individuals referred for bariatric surgery.

Recent studies found that in individuals with obesity, each component of MS added to obesity increased the risk of steatosis exponentially (31,32). Several risk factors have been associated with the development and progression of NAFLD and NASH in this population, most notably hypertension, diabetes and dyslipidemia (32,33). In previous studies with people with obesity diagnosed with NAFLD, there was a high prevalence of individuals with hypertension and dyslipidemia, but there was no association between NAFLD and T2DM (33,34). This was corroborated by the present study, where NAFLD and T2DM were present concomitantly in only 12% of cases.

Previous studies found a significant prevalence of hypertension among people with obesity, NAFLD and NASH (33,35,36), and a positive association between dyslipidemia, NAFLD and NASH in this same group of patients (37). Although these studies showed an association between hypertension and dyslipidemia and the development of NAFLD and its progression to NASH, we failed to find this association in this study.

In a previous study with 325 patients submitted for bariatric surgery (9), in agreement with the present study, the prevalence of individuals with diabetes was not very high, possibly due the predominance of young patients in the study populations. In the first study, 19% of the population presented with T2DM, and the mean patient age was 36 years. In the present study, only 12% had diabetes. This lower incidence may be related to the predominance of younger patients in the current study.

In this study, multivariate analysis revealed that age > 28 years and HOMA-IR ≥ 2.5 were significantly associated with the development of NAFLD, indicating that a significant number of people with obesity aged 28 years or over developed NAFLD when the HOMA index was greater than or equal to 2.5. Nevertheless, age ≥ 28 years has not been identified as a risk factor for developing NAFLD in other studies.

It is known that abnormal glucose metabolism is very common in patients with NAFLD (38). A study analyzed 152 individuals with obesity, with NAFLD and NASH and found that they had significant IR than healthy subjects (39), and in this study, multivariate analysis of the risk factors showed a significant association between the presence of NASH and AST/ALT < 1, increased triglycerides and low HDL-c. Patients with NAFLD also had significantly increased triglyceride levels, low HDL-c levels and AST/ALT < 1 (36), confirmed by other authors with similar findings (9). Moreover, some factors associated with the non progression of NAFLD to NASH were identified, such as the absence of SM and T2DM, normal ALT, AST, GGT and glycosylated hemoglobin and HOMA-IR > 3 (36). These findings are similar to the current study, which also showed that AST/ALT > 1 and normal ALT, cholesterol, triglycerides and GGT were associated with the absence of SM or dyslipidemia.

In our series, ALT levels were normal in 77% of patients without NASH, while in a similar study with 542 individuals with obesity with NASH, most of the population had elevated ALT levels (40). However, in the same study, we also noted an association between elevated ALT levels and the severity of liver involvement, which was not observed in the current study, due to minor liver involvement.

Abdominal ultrasound plays an important role in the diagnosis of NAFLD because it is a low-cost, noninvasive, readily available test, although it is not as accurate in individuals with obesity because of technical difficulties caused by the greater amounts of abdominal fat in these individuals (41,42). When used to diagnose NAFLD in people with obesity, ultrasound has a sensitivity and specificity of 49% and 75% respectively, in the study by Mottin and cols. (43). However, Leivas and cols. (44) found the sensitivity and specificity of 88.9% and 44.6% respectively, difference justified by the authors as being related to the number of patients with a negative biopsy result included in that study, low to estimate ultrasound specificity. We found 66.5% agreement of the results of the biopsy and the abdominal ultrasound, with sensitivity of 66% (61%-71%) and specificity of 85% (65%-100%) for ultrasound considering biopsy as gold standard. Sensitivity was greater than Mottin but minor than Leivas. Specificity was greater than both studies, but was estimated considering only 13 cases indicating low precision.

The gold standard for the diagnosis of NAFLD and NASH is liver biopsy (33,45), and there is a dearth of studies that define a universal clinical and histological protocol for predicting the course of NASH (9). In a study with 551 people with obesity, an average age of 36 years and biopsy availability, it was found that fibrosis was highly related (46,47).

Another study found that of 129 individuals with obesity that were availability of liver biopsy, only 26% had NAFLD; however, 55% of these patients had NASH and 31% had some degree of liver fibrosis. Among those with liver fibrosis, only 6.9% had moderate to severe fibrosis (F2/F3/F4), and one patient (0.7%) had cirrhosis (48). In the actual study, of the 415 patients with NAFLD, 26% had NASH. Of the patients with NAFLD, 42% had some degree of fibrosis (F1/F2/F3/F4), 21% had a moderate to severe degree (F2/F3/F4) and two patients (0.5 %) had cirrhosis. The cases with liver cirrhosis, but not diagnosed with NASH could be explained because steatosis become inconspicuous in cirrhosis and all histologic features of NASH, such as steatosis, ballooning, and Mallory-Denk bodies may not be evident when progress to cirrhosis (49). This condition without NASH evidence may be diagnosed as cryptogenic cirrhosis (49) and epidemiologic studies indicate that NASH is a common cause of cirrhosis clinically described as cryptogenic (50).

Caldwell and cols. (51) retrospectively reviewed the biopsy specimens from cirrhotic patients without sufficient histologic features to diagnose NASH but prior histologically confirmed noncirrhotic NASH. They found that macrovesicular steatosis, although uniformly present in the precirrhotic NASH specimens, declined in the late-stage cirrhotic NASH specimens and was not useful in the distinction of NASH cirrhosis from cirrhosis secondary to chronic viral hepatitis. Lai and cols. (50) found that Features supporting a steatohepatitic etiology include identification of ballooned hepatocytes and perisinusoidal fibrosis despite the presence of only minimal steatosis (50). Some conditions associated with NAFLD are described in the literature that can contribute to the evolution to cirrhosis, such as T2DM and obesity (49).

The low percentage of cases with severe fibrosis in our study may be because the population studied was predominantly young. In these individuals, bariatric surgery may prevent future permanent liver damage.

As a limitation of this study, there was the selection bias of the convenience sample, only patients and the retrospective nature of the study. Furthermore, the inclusion of a relatively young population may have influenced the classic association of the presence of T2DM and NASH, which was not observed in the present study. The liver biopsy was surgical, not performed by needle, so the sample may not have been very representative of the histological changes. Only patients with criteria to undergo bariatric surgery were studied, therefore it is not possible to extend the results of this study to all individuals with obesity who do not meet the criteria for bariatric surgery.

In conclusion, the majority of patients evaluated in this study had NAFLD (95%), but only 26% were diagnosed with NASH. Of these, 70% had mild or no liver fibrosis, 30% had moderate or advanced fibrosis (F2 or F3) or both, and none had cirrhosis. There was a significant prevalence of NAFLD in patients with HOMA-IR ≥ 2.5 and those over 28 years of age. The prevalence of NASH was significantly higher in patients with low HDL-c, high TG, and AST/ALT ≤ 1.
